# A Robust Localization Algorithm Based on NLOS Identification and Classification Filtering for Wireless Sensor Network

**DOI:** 10.3390/s20226634

**Published:** 2020-11-19

**Authors:** Long Cheng, Sihang Huang, Mingkun Xue, Yangyang Bi

**Affiliations:** 1 Department of Computer and Communication Engineering, Northeastern University, Qinhuangdao 066004, China; sihanghuang96@163.com (S.H.); mingkunxue61@gmail.com (M.X.); 2 SANY GROUP CO., Ltd., Changping, Beijing 102202, China; biyy@sany.com.cn

**Keywords:** wireless sensor network, NLOS identification, classification filtering, NLOS localization

## Abstract

With the rapid development of information and communication technology, the wireless sensor network (WSN) has shown broad application prospects in a growing number of fields. The non-line-of-sight (NLOS) problem is the main challenge to WSN localization, which seriously reduces the positioning accuracy. In this paper, a robust localization algorithm based on NLOS identification and classification filtering for WSN is proposed to solve this problem. It is difficult to use a single filter to filter out NLOS noise in all cases since NLOS cases are extremely complicated in real scenarios. Therefore, in order to improve the robustness, we first propose a NLOS identification strategy to detect the severity of NLOS, and then NLOS situations are divided into two categories according to the severity: mild NLOS and severe NLOS. Secondly, classification filtering is performed to obtain respective position estimates. An extended Kalman filter is applied to filter line-of-sight (LOS) noise. For mild NLOS, the large outliers are clipped by the redescending score function in the robust extended Kalman filter, yielding superior performance. For severe NLOS, a severe NLOS mitigation algorithm based on LOS reconstruction is proposed, in which the average value of NLOS error is estimated and the measurements are reconstructed and corrected for subsequent positioning. Finally, an interactive multiple model algorithm is employed to obtain the final positioning result by weighting the position estimation of LOS and NLOS. Simulation and experimental results show that the proposed algorithm can effectively suppress NLOS error and obtain higher positioning accuracy when compared with existing algorithms.

## 1. Introduction

A wireless sensor network (WSN) is a self-organized network consisted of a large number of inexpensive sensor nodes, which is increasingly widely applied in industrial production and social life [1]. Localization technology is one of the core technologies of WSN. WSN localization technology usually uses time of arrival (TOA), angle of arrival (AOA), time difference of arrival (TDOA), received signal strength (RSS) or other measurements to estimate the location of nodes [2,3]. In WSN localization, the signal transmission mode can be divided into two types, one is line-of-sight (LOS) transmission, in which the signal can be transmitted from one node to another in a straight path, and the other is non-line-of-sight (NLOS) transmission, in which the signal can only be transmitted between nodes by reflection or refraction due to obstruction of obstacles [4]. In the NLOS case, the signal transmission path will be longer than that in LOS case due to reflection or refraction, which will result in a larger positive transmission distance error, called NLOS error. Under usual circumstances, for LOS transmission we can use some effective algorithms such as extended Kalman filter (EKF) to achieve accurate positioning. However, LOS transmission is only an ideal and unrealistic assumption. In many real scenes, NLOS transmission is more common, which will obviously reduce the positioning performance. Mitigating NLOS error is the main goal of NLOS localization.

In order to obtain the accurate position of the target node, many algorithms have been proposed to solve the localization problem. The EKF [5] algorithm performs well in LOS, but its performance will be seriously affected when it is interfered with by NLOS. In an interactive multiple model (IMM) algorithm [6], a second-order Markov transition model is introduced to achieve switching between LOS and NLOS models. Two parallel filters are designed to filter LOS and NLOS respectively, and the final result is fused with the position estimation of the two filters. Based on some prior statistical knowledge of NLOS error, the IMM algorithm has better performance in LOS/NLOS mixed positioning environment, but the prior statistical knowledge of NLOS error is usually unknown. On the basis of the IMM algorithm, by introducing robust technology to solve the EKF equation, robust extended Kalman filter (REKF) is proposed to replace the NLOS filter in the IMM algorithm, and furthermore a robust IMM algorithm (R-IMM) [7] is developed. This algorithm does not assume statistical knowledge of NLOS errors and only assumes that sensor noise variance is known. However, the clipping parameters $c_{1}$ and $c_{2}$ for REKF in R-IMM algorithm need to be set manually, which is difficult and inflexible when dealing with complicated NLOS environment, and R-IMM algorithm is inappropriate when the NLOS probability is large (larger than 0.6). The above algorithms all have a common shortcoming: either they are based on some priori knowledge of NLOS error which is unknown in fact, or their robustness is weak in a severe NLOS environment, which means that they can only perform well in a LOS or mild NLOS environment. For the traditional NLOS localization algorithms, the main challenge of eliminating NLOS error is how to achieve robust localization in the severe NLOS environment without assuming the prior statistical knowledge of NLOS error. The motivation of our work is to solve these problems. In this paper, a robust localization algorithm based on NLOS identification and classification filtering (NI-CF) for WSN is proposed using the TOA measurement, the main contributions of this paper are as follows:

1. The NLOS identification strategy is proposed to detect the severity of NLOS and further divide the NLOS situation into mild NLOS and severe NLOS. Compared with the traditional algorithms, the proposed algorithm can obtain more NLOS environment information through NLOS identification, which is beneficial to improve the positioning accuracy and robustness of the algorithm.
2. The proposed algorithm has strong robustness even in a severe NLOS environment. A severe NLOS mitigation algorithm based on LOS reconstruction is proposed to deal with severe NLOS. When severe NLOS occurs, the average value of NLOS error is estimated and the measurements are reconstructed and corrected for subsequent positioning, so our algorithm has better performance in severe NLOS environment when compared with the traditional algorithms.
3. By contrast with many algorithms, the proposed algorithm does not assume the prior information of NLOS error. Even so, simulation and experimental results show that the proposed algorithm still effectively eliminates NLOS error when NLOS error obeys different distributions. Without losing generality, the proposed algorithm uses TOA measurement, but it can be extended easily to other signal features such as TDOA, RSS or AOA.
4. The clipping parameters $c_{1}$ and $c_{2}$ for REKF in the proposed algorithm do not need to be frequently manually adjusted according to different positioning environments since REKF is determined to filter mild NLOS instead of all NLOS as in RIMM, which broadens the application scenarios of the algorithm.

The organizational structure of the remaining papers is as follows. The related works and problem statement are described in Section 2 and Section 3, respectively. The proposed method is introduced in detail in Section 4 and the simulation and experimental results are presented in Section 5. Section 6 draws the conclusion.

## 2. Related Works

In order to reduce the influence of NLOS transmission, many scholars have carried out a series of studies. Haigh proposed a novel NLOS ultrasonic signal classification and rejection algorithm [8], which uses the amplitude and residual of the received signal as two features to classify the signal and reject the NLOS signal. Experiments show that the algorithm can correctly classify 96% of ultrasonic signals with a lower computational cost, but the accuracy of the TOF estimation needs further improvement. In [5], Gaussian mixture model, EKF and IMM are integrated to overcome the influence of frequent switching of signal transmission between LOS environment and NLOS environment. The main advantage of the algorithm is that it can weaken the influence of severe NLOS in the mixed environment of LOS/NLOS, but some knowledge of the noise statistics is required in this algorithm, which is actually unknown. A frequency-dependent transfer function is constructed in [9] to transform the propagation channel in the real scene into the propagation channel in free space, further eliminating the NLOS effect. Simulation results show that the algorithm has higher positioning accuracy than the traditional TDOA method and does not depend on the form of signal. However, a positioning experiment in a real scene is needed to verify the performance of the algorithm. Some linear regression estimates are proposed in [10,11], and the authors in [10] explore the asymptotic modified M-estimators (MM-estimators), and Kalina proposes new weighing schemes together with corresponding estimates for the variance of disturbances by focusing on estimating the variance of the random regression errors in [11]. Wang proposes an RSS-based localization algorithm [12], whose main idea is to estimate the LOS components from fading signals, and then use the LOS signals to calculate the positioning result. The advantage of this algorithm is that it is robust to environmental fading disturbance, and it performs better than traditional RSS-based methods, but this algorithm needs to be compared with more algorithms. Through small error analysis, Picard establishes an expression model of NLOS error and covariance in [13] to counter the multipath effect of signal propagation. This algorithm can effectively handle multipath which the traditional direct position determination algorithm cannot cope with well. However, this algorithm is based on some assumptions for generic scenarios, which needs more practical verification.

Data fusion is also one of the research hotspots. In [14], by combining the measurements of TOA and AOA, Wu proposes a probability weighting localization algorithm, in which range and angle measurements are divided into different combinations and corresponding probabilities are calculated to weight the results of these combinations. This algorithm performs better than existing algorithms, but its computational complexity is higher. In [15], Chang transforms the positioning problem into a second-order cone programming problem to solve by using the combined measurements of AOA and RSS, which realizes accurate positioning under the conditions of known and unknown target node transmission power. However, the superior performance is at the cost of higher computational complexity, which makes it difficult for the algorithm to be applied to computers with poor computing power. Based on combined TOA and RSS measurements, Tomic converts the localization problem into a generalized trust region sub-problem [16], which is then solved by a bisection procedure. The main idea of the algorithm is to eliminate NLOS error as a noise parameter. The advantage of this algorithm is its linear computational complexity, but it is only suitable for small noise power. In [17], Coluccia proposes that the mixed measurement of RSS and TOA does not always benefit the estimator. In the vicinity of a critical distance, the combined RSS-TOA measurement is not as good as the RSS and TOA measurement alone. To solve this problem, a heuristic approach to choose the optimal measurements for each link is proposed in [18], which effectively uses the measurements of RSS and TOA. The algorithm does not depend on the knowledge of the noise powers. However, only the exponential distribution simulation is carried out in this paper, which cannot fully cover the complex situation in real world. In [19], by combining TOA and RSS measurements, Chen designs a position estimator based on the idea of combining IMM algorithm and EKF filter, which effectively enhances the positioning precision in hybrid LOS and NLOS environments. Nevertheless, this algorithm also depends on the knowledge of the NLOS error statistics.

Many conventional algorithms need to know the prior information of the environment in advance. How to reduce the dependence on the prior information of the environment is also one of the research directions. In [20], Yu establishes a range selection rule using the identified channel information to select the measurements, which can reduce the NLOS error initially. After that, robustness is introduced into Taylor series least square method to further mitigate the remaining NLOS error. The highlight of this algorithm is that it does not assume the threshold and distribution function of NLOS error, but its performance depends on a large number of offline collected data. Without knowing the prior information of specific channel state and NLOS error statistics, the paper [21] establishes a balance parameter to solve the problem that the traditional robust algorithm cannot perform well in a sparse NLOS environment. The localization problem is then converted into non-convex programming in order to solve it. Simulation results show that it works well for both the spare and dense NLOS environments. However, its computational complexity is higher, which limits its application scenarios. By applying the robust statistics [22,23] in a multiple model framework, a robust IMM algorithm (R-IMM) is proposed in [7] to reduce the NLOS error. The advantage of this algorithm is that it performs well in the mixed environment of LOS/NLOS, and it does not assume any statistical information of the NLOS error. However, the clipping parameters $c_{1}$ and $c_{2}$ for REKF in this algorithm need to be set manually, which is difficult and inflexible when dealing with a complicated NLOS environment, and it is inappropriate when the NLOS probability is large (larger than 0.6). For cooperative localization, the information of nodes (agents) whose positions have not been determined is also helpful for positioning, but the positions of agents are unknown. Mendrzik proposes a position-constrained inference algorithm in [24] to constrain the positions of the agents in a limited area, which improved the positioning accuracy. This algorithm does not need the prior position information of the agents and it has lower computational complexity. Nevertheless, its robustness needs to be further improved.

## 3. Problem Statement

### 3.1. Signal Model

**Figure 1:**
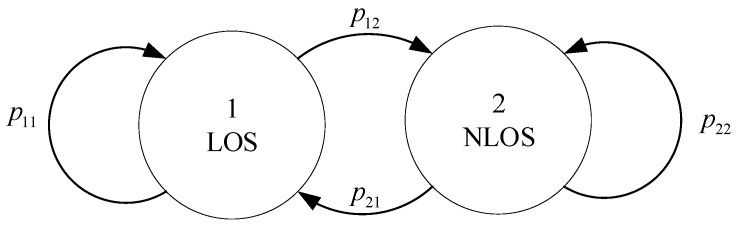
Markov switching model.

In a two-dimensional localization area, we assume that there is one mobile node surrounded by *M* beacon nodes whose coordinates are known, and the signal transmission between the mobile node and the beacon nodes can be LOS or NLOS depending on whether there are obstacles between the mobile node and the beacon nodes. The mobile node moves randomly in the localization area. In the process of mobile node moving, the signal transmission channels between the mobile node and the beacon nodes switch between LOS and NLOS conditions, and the switching is regarded as a second-order Markov transition process [19] as shown in Figure 1. The state vector of the mobile node is denoted as $\theta \left(l\right)={\left[\begin{array}{cccc} x\left(l\right) & y\left(l\right) & \dot{x}\left(l\right) & \dot{y}\left(l\right) \end{array}\right]}^{T}$, where (x(l),y(l)) represents the position of the mobile node and $\left(\dot{x}\left(l\right),\dot{y}\left(l\right)\right)$ represents the speed of the mobile node at time step *l*. The movement of the mobile node can be described by the change of the state vector, which can be modeled as:

(1) $$\begin{array}{ccc} \theta \left(l\right)=A\theta \left(l-1\right)+G\omega \left(l-1\right) & & l=1,2,\ldots ,L \end{array}$$

(2) $$\begin{array}{cc} A=\left[\begin{array}{cccc} 1 & 0 & \Delta t & 0\\ 0 & 1 & 0 & \Delta t\\ 0 & 0 & 1 & 0\\ 0 & 0 & 0 & 1 \end{array}\right] & G=\left[\begin{array}{cc} \Delta t^{2}/2 & 0\\ 0 & \Delta t^{2}/2\\ \Delta t & 0\\ 0 & \Delta t \end{array}\right] \end{array}$$

where, Δt is the sampling period, the driving noise ω(l) is assumed to be Gaussian white noise with a mean of zero and a covariance matrix of Q(l). *A* is the state transition matrix of the mobile node, and *G* is used to describe the random acceleration caused by noise ω(l).

Let $D(l)={\left[d_{1}(l),d_{2}(l),\ldots ,d_{m}(l),\ldots ,d_{M}(l)\right]}^{T}$ denote the range measurements based on TOA data between the mobile node and *M* beacon nodes at time step *l*. The element $d_{m}(l)$ represents the range measurement between the *m*-th beacon node and the mobile node. Then,

(3) D(l)=h(θ(l))+v(l)

where $h(\theta (l))=[h_{1}(\theta (l)),h_{2}(\theta (l)),\ldots ,h_{m}(\theta (l)),\ldots ,h_{M}(\theta (l)){]}^{T}$, and $h_{m}(\theta (l))$ represents the true Euclidean distance between the *m*-th beacon node and the mobile node at time step *l*, which is defined as:

(4) $$\begin{array}{cc} h_{m}\left(\theta \left(l\right)\right)=\sqrt{{\left(x\left(l\right)-x_{BS,m}\right)}^{2}+{\left(y\left(l\right)-y_{BS,m}\right)}^{2}} & m=1,2,\ldots ,M \end{array}$$

where $\left(x_{BS,m},y_{BS,m}\right)$ represents the two-dimensional coordinate of the *m*-th beacon node. Noise vector v(l) describes sensor noise and NLOS error. Sensor noise is modeled as Gaussian white noise with a mean of zero and a variance of $\sigma_{G}^{2}$, and the prior distribution information of NLOS error is unknown. The measurement covariance matrix:

(5) $$R(l)=E\{(v(l)-E\{v(l)\}){\left(v(l)-E\{v(l)\}\right)}^{T}\}$$

is defined as,

(6) $$R(l)=diag[\sigma_{1}{}^{2},\sigma_{2}{}^{2},\ldots ,\sigma_{m}{}^{2},\ldots \sigma_{M}{}^{2}]$$

where, the element $\sigma_{m}$ is defined as,

(7) $$\sigma_{m}=\{\begin{array}{cc} \sigma_{G} & if\;LOS\;condition\\ \sqrt{\sigma_{G}^{2}+\sigma_{NLOS}^{2}} & if\;NLOS\;condition \end{array}$$

We assume that the sensor noise variance $\sigma_{G}^{2}$ is known, and the true measurement noise covariance matrix R(l) is unknown since the prior distribution information of NLOS error is unknown. Instead, $R^{*}(l)$ denotes the measurement noise covariance matrix set by the tracker.

### 3.2. A Brief Introduction to the Interactive Multiple Model (IMM)

The IMM localization algorithm regards the signal propagation channel as a second-order Markov transition process [19]. Four main steps are included in the algorithm. The first step is input interaction. In this step, the mixed probability is calculated to obtain the mixed covariance matrix and state estimation. The second step is to carry out model matching. Two filters are designed for the LOS model and NLOS model to obtain two estimation results, which should be able to handle LOS and NLOS cases, respectively, well. The third step is mode probability update. The new mode probability is updated by a calculated likelihood function. The last step is combination. Based on their respective mode probabilities, the final positioning results are estimated by combining the estimation of LOS and NLOS model. Because it combines the localization results of LOS and NLOS models, the IMM algorithm is suitable for localization in the hybrid situation of LOS and NLOS.

## 4. Proposed Method

### 4.1. General Concept

**Figure 2:**
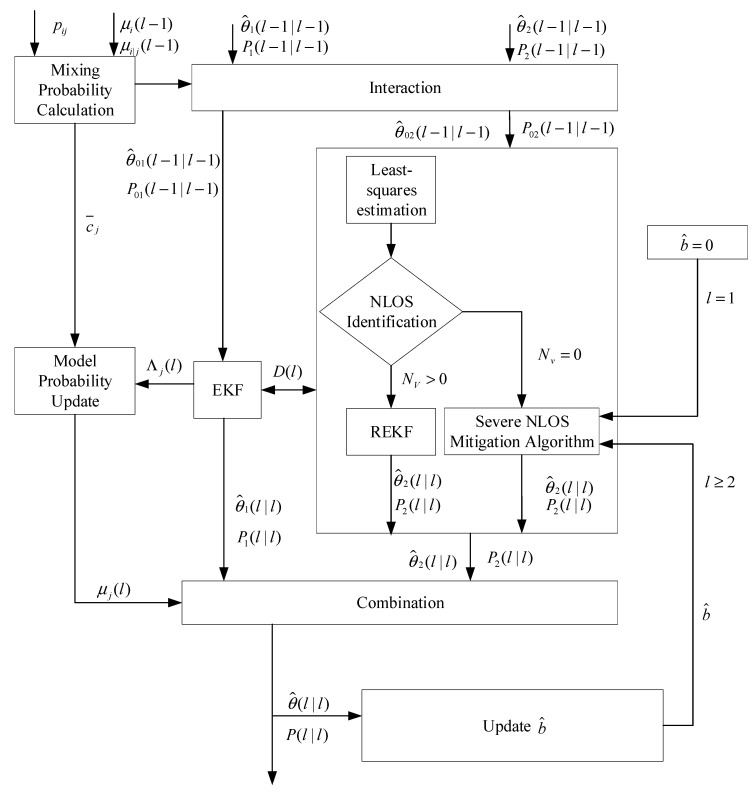
The flowchart of the proposed algorithm.

The flowchart of the proposed algorithm is illustrated in Figure 2. Firstly, a NLOS identification strategy is proposed to detect the severity of NLOS. In this strategy, the measurements are first divided into *N* different subgroups, and each subgroup separately calculates its own position estimation by the least-squares, and then NLOS identification is carried out on *N* different position estimation results using the validation gate. The position estimation that can fall into the validation gate is a positioning estimation with higher positioning accuracy. We count the number $N_{v}$ of position estimates that fall into the validation gate. $N_{v}$ can be used to characterize the severity of NLOS. The larger $N_{v}$, the milder the degree of NLOS, and the smaller $N_{v}$, the severer the degree of NLOS. Then NLOS situations can be divided into two categories according to the severity: mild NLOS and severe NLOS. If $N_{v}>0$, there is position estimation that can fall into the validation gate, and it means that NLOS is not particularly serious, which is classified as mild NLOS. If $N_{v}=0$, no position estimation falls into the validation gate, it indicates that the NLOS is extremely serious at this time, which is classified as severe NLOS. Secondly, classification filtering, which combines EKF, REKF and the proposed severe NLOS mitigation algorithm based on LOS reconstruction, is performed to obtain respective position estimates. EKF and REKF are applied to filter LOS noise and mild NLOS noise respectively. Meanwhile, a severe NLOS mitigation algorithm based on LOS reconstruction is proposed to filter out severe NLOS noise. Finally, the IMM algorithm is employed to obtain the final positioning result by weighting the position estimation of LOS and NLOS.

### 4.2. Interaction and Prediction

First, the state vector $\hat{\theta }\left(l-1|l-1\right)$, covariance matrix P(l−1|l−1) at time step *l*-1 are initialized respectively [7]. Then, we need to calculate the mixed probability $\mu_{i|j}(l-1|l-1)$, which can be obtained by the following formula:

(8) $$\begin{array}{cc} \mu_{i|j}(l-1|l-1)=(1/{\bar{c}}_{j})p_{ij}\mu_{i}(l-1) & l=1,\ldots ,L \end{array}$$

where, $\mu_{i}(l-1)$ is the mode probability of the *i*-th mode. $p_{ij}$ is Markov transition probability from mode *i* to mode *j*. Note that i,j ε {1 for LOS mode, 2 for NLOS mode}. ${\bar{c}}_{j}$ denotes the normalization factor, which is

(9) $${\bar{c}}_{j}=\sum_{i}p_{ij}\mu_{i}(l-1)$$

Then input interaction is carried out:

(10) $${\hat{\theta }}_{0j}\left(l-1|l-1\right)=\sum_{i}{\hat{\theta }}_{i}\left(l-1|l-1\right)\mu_{i|j}(l-1|l-1)$$

(11) $${\tilde{\theta }}_{ij}\left(l-1|l-1\right)={\hat{\theta }}_{i}\left(l-1|l-1\right)-{\hat{\theta }}_{0j}\left(l-1|l-1\right)$$

(12) $$P_{0j}\left(l-1|l-1\right)=\sum_{i}\mu_{i|j}(l-1|l-1)\{P_{i}\left(l-1|l-1\right)+{\tilde{\theta }}_{ij}\left(l-1|l-1\right).{\tilde{\theta }}_{ij}{}^{T}\left(l-1|l-1\right)\}$$

where, ${\hat{\theta }}_{0j}\left(l-1|l-1\right)$ and $P_{0j}\left(l-1|l-1\right)$ are the mixed state estimation and mixed covariance matrix for the *j*-th mode-matched filter at time step *l* − 1. After the interaction is completed, the algorithm starts to perform model matching.

Prediction: through the interaction process, we obtain the mixed state estimation and the mixed state covariance matrix at time step *l* − 1, which are employed to compute the state prediction value and the error covariance matrix prediction value for LOS/NLOS at time step *l*.

(13) $${\hat{\theta }}_{j}\left(l|l-1\right)=A{\hat{\theta }}_{0j}\left(l-1|l-1\right)$$

(14) $$P_{j}\left(l|l-1\right)=AP_{0j}\left(l-1|l-1\right)A^{T}+CQ(l)C^{T}$$

### 4.3. A Non-Line-of-Sight (NLOS) Identification Strategy

Before suppressing NLOS noise, a NLOS identification strategy is proposed to detect the severity of NLOS.

Since the position estimation requires at least 3 measurement values, we first divide the distance measurement values of *M* beacon nodes and mobile node into $N=\left(\begin{array}{c} M\\ 3 \end{array}\right)$ different subgroups according to 3 measurement values in each group. For each subgroup, the corresponding position estimation $z_{n}(l)$ is obtained through least square estimation. The position estimation prediction value $\hat{z}(l|l-1)$ of the mobile node is calculated as follows:

(15) $$\hat{z}(l|l-1)=B{\hat{\theta }}_{2}(l|l-1)$$

(16) $$\begin{array}{cc} v_{2,n}(l)=z_{n}(l)-\hat{z}(l|l-1) & n=1,\ldots ,N \end{array}$$

where, observation matrix $B=\left[\begin{array}{cccc} 1 & 0 & 0 & 0\\ 0 & 1 & 0 & 0 \end{array}\right]$, and $v_{2,n}(l)$ is the innovation of the *n*-th subgroup at time step *l.* After calculating the position estimation of each subgroup, we conduct NLOS identification to detect the severity of NLOS pollution.

When there is no NLOS error in all range measurements of the subgroup, then the following formula holds:

(17) $$\begin{array}{cc} v_{2,n}(l)∼N(0,S_{2,n}(l)) & n=1,\ldots ,N \end{array}$$

where, $S_{2,n}(l)$ represents the innovation covariance matrix of the *n*-th subgroup at time step *l*, calculated as follows:

(18) $$S_{2,n}(l)=BP_{2}(l|l-1)B^{T}+\sigma_{G}{}^{2}{\left(H^{T}{}_{2,n}(z_{n})H_{2,n}(z_{n})\right)}^{-1}$$

(19) $$H_{2,n}\left(z_{n}\right)=\frac{\partial h_{2,n}({\left[x,y\right]}^{T})}{\partial [x,y]}{|}_{x=\hat{x},y=\hat{y}}$$

To test Equation (17), we define hypothesis $\zeta_{0,n}$ and alternative hypothesis $\zeta_{1,n}$:

(20) $$\begin{array}{cc} \zeta_{0,n}:v_{2,n}(l)\sim N(0,S_{2,n}(l)) & n=1,\ldots ,N \end{array}$$

(21) $$\begin{array}{ccc} \zeta_{1,n}:\mathrm{not} & \zeta_{0,n} & n=1,\ldots ,N \end{array}$$

If all beacon nodes of the *n*-th subgroup are in LOS condition, the position estimation $z_{n}(l)$ can fall into the validation gate, so the hypothesis $\zeta_{0,n}$ holds. If at least one beacon node of the *n*-th subgroup is in NLOS condition, the position estimation $z_{n}(l)$ is wrong and a higher innovation covariance will be generated, then the hypothesis $\zeta_{1,n}$ holds, and $z_{n}(l)$ fall outside the validation gate. In order to evaluate whether $z_{n}(l)$ can fall into the validation gate, we calculate the test statistic $T_{n}(l)$ as follows:

(22) $$T_{n}(l)=v^{T}{}_{2,n}(l)S^{-1}{}_{2n}(l)v_{2,n}(l)$$

By comparing $T_{n}(l)$ with the threshold of the validation gate γ, we can verify whether the position estimation $z_{n}(l)$ can fall into the validation gate. If $T_{n}(l)$ is smaller than γ, then $z_{n}(l)$ can fall into the validation gate, the hypothesis $\zeta_{0,n}$ holds. If $T_{n}(l)$ is larger than γ, then $z_{n}(l)$ falls outside the validation gate, the hypothesis $\zeta_{1,n}$ holds. The threshold γ of the validation gate is related to the threshold probability $P_{G}$, which denotes the probability that $z_{n}(l)$ computed from the LOS beacon nodes falls into the validation gate. $P_{G}$ is

(23) $$P_{G}=\int_{0}^{\gamma }f_{\chi 2(2)}(x)dx=1-P_{FA}$$

where $f_{\chi 2(2)}(\cdot )$ denotes the chi-square distribution probability density function, which has two degrees of freedom, and $P_{FA}$ is warning probability. When probability $P_{G}$ is determined, we can obtain the value of threshold γ through a chi-square distribution table.

We count the number of $z_{n}(l)$ that can fall into the validation gate, and record it as $N_{v}$$\left(0\leq N_{v}\leq N\right)$. Then, NLOS situations can be divided into two categories according to the severity: mild NLOS and severe NLOS.

If $N_{v}=N$, it means that beacon nodes of the all subgroups are in LOS condition, and there is no NLOS error at this time. We will not consider this situation in the NLOS model.

If $0<N_{v}<N$, the position estimation $z_{n}(l)$ of some subgroups can fall into the validation gate, while the position estimation $z_{n}(l)$ of another subgroups is polluted by NLOS, but the degree of NLOS is not extremely serious, because the position estimation $z_{n}(l)$ of some subgroups still falls into the validation gate. This condition is classified as mild NLOS.

If $N_{v}=0$, there is no position estimation $z_{n}(l)$ of subgroup that can fall into the validation gate, which shows that at least one beacon node in each subgroup is polluted by NLOS. This condition is classified as severe NLOS.

### 4.4. Classification Filtering

Update: when *j* = 1, EKF filter is used to update the state estimation and error covariance matrix.

(24) $$H_{1}\left(l\right)=\frac{\partial h(\theta (l))}{\partial \theta (l)}{|}_{\theta (l)={\hat{\theta }}_{1}(l|l-1)}$$

(25) $$v_{1}(l)=D(l)-h({\hat{\theta }}_{1}(l|l-1))$$

(26) $$S_{1}(l)=H_{1}(l)P_{1}(l|l-1)H_{1}{}^{T}(l)+R_{1}{}^{*}(l)$$

where, $H_{1}\left(l\right)$ and $v_{1}(l)$ are the Jacobian matrix and innovation at time step *l*, $R_{1}^{*}(l)$ is the covariance matrix for LOS model, and $S_{1}(l)$ denotes the innovation covariance matrix.

(27) $$K_{1}(l)=P_{1}(l|l-1)H_{1}{}^{T}(l)S_{1}{}^{-1}(l|l-1)$$

(28) $${\hat{\theta }}_{1}(l|l)={\hat{\theta }}_{1}(l|l-1)+K_{1}(l)v_{1}(l)$$

(29) $$P_{1}(l|l)=(I_{4}-K_{1}(l)H_{1}(l))P_{1}(l|l-1)$$

(30) $$\wedge_{1}(l)=N(v_{1}(l);0,S_{1}(l))$$

where, $K_{1}(l)$ represents the extended Kalman gain, $\wedge_{1}(l)$ is the likelihood function. ${\hat{\theta }}_{1}(l|l)$ and $P_{1}(l|l)$ are the updated state estimation and the updated error covariance matrix at time step *l.*

When *j* = 2, the REKF algorithm and a proposed severe NLOS mitigation algorithm based on LOS reconstruction are employed to filter mild NLOS and severe NLOS, respectively.

### 4.5. Robust Extended Kalman Filter (REKF) Algorithm

The REKF algorithm is described as follows:

(1) Rewrite EKF equation: it was proved in available research works [23,25,26] that the EKF filter can be transformed into the least square solution of linear regression problem. In order to apply robust technology, we transform EKF equation into linear regression model. Before that, we first calculate the Jacobian matrix $H_{2}(l)$, the innovation $v_{2}(l)$ and the innovation covariance matrix $S_{2}(l)$ at time step *l.*

(31) $$H_{2}\left(l\right)=\frac{\partial h(\theta (l))}{\partial \theta (l)}{|}_{\theta (l)={\hat{\theta }}_{2}(l|l-1)}$$

(32) $$v_{2}(l)=D(l)-h({\hat{\theta }}_{2}(l|l-1))$$

(33) $$S_{2}(l)=H_{2}(l)P_{2}(l|l-1)H_{2}{}^{T}(l)+R_{2}{}^{*}(l)$$

Then, the state equation and the measurement equation in (1) and (3) are rewritten as follows:

(34) $$\left[\begin{array}{c} I_{4}\\ H_{2}(l) \end{array}\right]x(l)=\left[\begin{array}{c} A{\hat{\theta }}_{02}(l-1|l-1)\\ D(l)-h({\hat{\theta }}_{2}(l|l-1)+H_{2}(l){\hat{\theta }}_{2}(l|l-1) \end{array}\right]+e(l)$$

where,

(35) $$e(l)=\left[\begin{array}{c} A(\theta (l-1)-{\hat{\theta }}_{02}(l-1|l-1))+G\omega (l-1)\\ -v(l) \end{array}\right]$$

At the same time, e(l) satisfies:

(36) $$E(e(l)e^{T}(l))=\left[\begin{array}{cc} P_{2}(l|l-1) & 0\\ 0 & R_{2}{}^{*}(l) \end{array}\right]=C(l)C^{T}(l)$$

where, C(l) is obtained by using Cholesky decomposition for $E(e(l)e^{T}(l))$. Then, the Equation (34) is multiplied by $C^{-1}(l)$ to obtain the linear regression model.

(37) $$\tilde{y}=F\theta +b\tilde{f}v+\tilde{v}$$

where

(38) $$\tilde{y}=C^{-1}(l)\left[\begin{array}{c} {\hat{\theta }}_{2}(l|l-1)\\ D(l)-h({\hat{\theta }}_{2}(l|l-1))+H_{2}(l){\hat{\theta }}_{2}(l|l-1) \end{array}\right]$$

(39) $$F=C^{-1}(l)\left[\begin{array}{c} I_{4}\\ H_{2}(l) \end{array}\right],\theta =\theta (l),\tilde{v}=-C^{-1}(l)e(l)$$

(2) Robust regression: considering the case of the linear regression model (37) at time step *l*, by solving the following coupled equation, we can obtain the maximum likelihood estimation of θ.

(40) $$\sum_{i=1}^{M+\dim(\theta )}{\left[F\right]}_{ij}\times \psi ({\tilde{y}}_{i}-\sum_{j\prime }^{\dim(\theta )}{\left[F\right]}_{ij^{\prime }}\theta_{j^{\prime }})=0,j^{\prime }=1,\ldots ,\dim(\theta )$$

where, dim(θ) denotes the dimension of θ and ψ is location score function. By solving Equation (37) by least-squares [26], we can calculate the state estimation ${\hat{\theta }}_{2}(l|l)={\left(F^{T}F\right)}^{-1}F^{T}\tilde{y}$, and the error covariance matrix $P_{2}(l|l)={\left(D^{T}D\right)}^{-1}$. However, the least-squares estimation is so vulnerable to outliers that we should adopt robust regression technique [27] to solve Equation (37). Since the NLOS error is always greater than zero, the probability density function of NLOS error is asymmetric, we use the descending score function [28] as the location score function as follows:

(41) $$\psi (v)=\{\begin{array}{cc} v & |v|\leq c_{1}\\ b\tanh[0.5b(c_{2}-|v|)]\mathrm{sgn}(v) & c_{1}\leq |v|\leq c_{2}\\ 0 & |v|>c_{2} \end{array}$$

where $c_{1}$ and $c_{2}$ are the clipping points of location score function, which are selected based on the efficiency loss we are willing to pay in the LOS condition. Choosing the appropriate b makes ψ(v) continuous at $c_{1}$. In this paper, $c_{1}$ and $c_{2}$ are set to 1.5 and 3, which are the same as those in [7]. Then, the state estimation of the mobile node is calculated by iterative Newton–Raphson steps based on the location score function. The process of the algorithm is as follows:

In the first step, an initial state estimation ${\hat{\theta }}_{2,}{}_{0}$ is computed by least-squares.

(42) $$\begin{array}{cc} {\hat{\theta }}_{2,}r={\left(F^{T}F\right)}^{-1}F^{T}\tilde{y} & r=0 \end{array}$$

where the index r denotes the *r*-th iteration.

In the second step, the initial state estimation ${\hat{\theta }}_{2,}{}_{0}$ is used to estimate the noise residual $\hat{V}$.

(43) $$\hat{V}=\tilde{y}-F{\hat{\theta }}_{2,r}$$

The third step is to estimate the noise scale ${\hat{\sigma }}_{V}$ after obtaining the noise residual $\hat{V}$.

(44) $${\hat{\sigma }}_{V}=1.48mad(\hat{V})$$

where mad(⋅) represents the mean absolute deviation and is defined as

(45) $$mad(\hat{V})=mean\left\{\left|\hat{V}-mean(\hat{V})\right|\right\}$$

The fourth step is to update the state estimation by a Newton–Raphson step. The new state estimation is computed as follows:

(46) $${\hat{\theta }}_{2,r+1}={\hat{\theta }}_{2,r}+\mu {\left(F^{T}F\right)}^{-1}F^{T}\psi (\hat{V}/{\hat{\sigma }}_{V})$$

where

(47) $$\mu =1/(1.25\max(|\psi^{\prime }(\hat{V}/{\hat{\sigma }}_{V})|))$$

In the fifth step, the convergence of the algorithm is checked according to a preset convergence condition. If the norm of the current state estimation and the last estimation is less than the required value, the iteration is stopped to obtain the final state estimation, otherwise, the above steps are repeatedly executed until convergence.

(48) $$\begin{array}{cc} ||{\hat{\theta }}_{2,r+1}-{\hat{\theta }}_{2,r}||<\varepsilon & \varepsilon >0 \end{array}$$

After obtaining the state estimation, the likelihood function is calculated as follows:

(49) $$\wedge_{2}(l)=N(v_{2}(l);0,S_{2}(l))$$

### 4.6. A Severe NLOS Mitigation Algorithm Based on Line-of-Sight (LOS) Reconstruction

The severe NLOS mitigation algorithm based on LOS reconstruction we propose is described as follows:

LOS reconstruction: When NLOS pollution is especially serious, if we can estimate the average value of NLOS error and perform LOS reconstruction on the measurements from each beacon node, the negative impact of NLOS error on positioning performance can be alleviated to a large extent. We first estimate the average value of NLOS error $\hat{b}$.

Update $\hat{b}$: we consider the position estimation at time step *l*. when l=1, we initialize $\hat{b}=0$. When l≥2, we use the positioning result before the current time to estimate the average value of NLOS error $\hat{b}$. The estimation value of NLOS error at time step *l* can be computed by using the position estimation $\hat{\theta }(l|l)$.

(50) $$\begin{array}{cc} b(l)=\frac{\sum_{m=1}^{M}d_{m}(l)-||{\left[\hat{\theta }(l|l)(1),\hat{\theta }(l|l)(2)\right]}^{T}-{\left[x_{BS,m},y_{BS,m}\right]}^{T}||}{M} & l=1,\ldots ,L-1 \end{array}$$

Since the NLOS error is a deviation greater than zero, we take the average value of all values larger than zero in the array [b(1),b(2),…,b(l−1)] as the estimated value $\hat{b}$ at time step *l*.

Distance reconstruction:

(51) $$D(l)={\left[d_{1}(l)-\hat{b},d_{2}(l)-\hat{b},\ldots ,d_{M}(l)-\hat{b}\right]}^{T}$$

Then the NLOS EKF filter is used to estimate the position using the measurements after LOS reconstruction.

(52) $$H_{2}\left(l\right)=\frac{\partial h(\theta (l))}{\partial \theta (l)}{|}_{\theta (l)={\hat{\theta }}_{2}(l|l-1)}$$

(53) $$v_{2}(l)=D(l)-h({\hat{\theta }}_{2}(l|l-1))$$

(54) $$S_{2}(l)=H_{2}(l)P_{2}(l|l-1)H_{2}{}^{T}(l)+R_{2}{}^{*}(l)$$

where, $H_{2}\left(l\right)$ and $v_{2}(l)$ denote the Jacobian matrix and innovation at time step *l*, $R_{2}{}^{*}(l)$ is the covariance matrix for NLOS model, and $S_{2}(l)$ denotes the innovation covariance matrix.

(55) $$K_{2}(l)=P_{2}(l|l-1)H_{2}{}^{T}(l)S_{2}{}^{-1}(l|l-1)$$

(56) $${\hat{\theta }}_{2}(l|l)={\hat{\theta }}_{2}(l|l-1)+K_{2}(l)v_{2}(l)$$

(57) $$P_{2}(l|l)=(I_{4}-K_{2}(l)H_{2}(l))P_{2}(l|l-1)$$

(58) $$\wedge_{2}(l)=N(v_{2}(l);0,S_{2}(l))$$

where, $K_{2}(l)$ represents the extended Kalman gain, $\wedge_{2}(l)$ is the likelihood function. ${\hat{\theta }}_{2}(l|l)$ and $P_{2}(l|l)$ are the updated state estimation and the updated error covariance matrix at time step *l*.

### 4.7. Model Probability Update

See below

(59) $$\mu_{j}(l)=(1/c)\wedge_{j}(l){\bar{c}}_{j}$$

(60) $$c=\sum_{j}\wedge_{j}(l){\bar{c}}_{j}$$

where $\mu_{j}(l)$ and *c* denotes the updated mode probability and the normalization factor respectively.

### 4.8. Combination

The output state vector estimation $\hat{\theta }(l|l)$ and error covariance matrix P(l|l) are:

(61) $$\hat{\theta }(l|l)=\sum_{j}{\hat{\theta }}_{j}(l|l)\mu_{j}(l)$$

(62) $$P(l|l)=\sum_{j}\{P_{j}(l|l)+[{\hat{\theta }}_{j}(l|l)-\theta (l|l)]\times {\left[{\hat{\theta }}_{j}(l|l)-\theta (l|l)\right]}^{T}\}\mu_{j}(l)$$

## 5. Simulation and Experimental Results

### 5.1. Simulation

#### 5.1.1. Simulation Environment and Parameter Settings

The simulation environment and parameter settings for simulation are introduced in this section.

**Figure 3:**
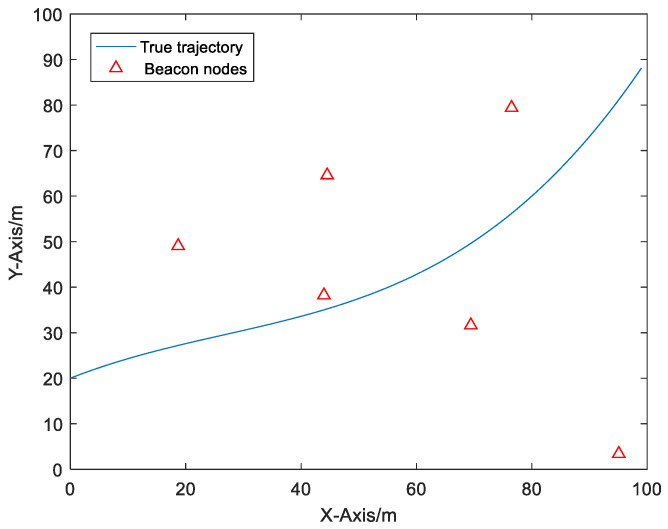
The simulation scene of a Monte Carlo experiment.

Firstly, we introduce the simulation environment. The simulation platform is MATLAB, and every simulation result is based on 1000 Monte Carlo experiments. The simulation scene of a Monte Carlo experiment is depicted in Figure 3. Note that the deployment of beacon nodes is not always the same in each Monte Carlo experiment. The positions of beacon nodes applied with Monte Carlo in each run are randomly deployed in a localization area of 100 m × 100 m. The purpose of random deployment of beacon nodes is to simulate the different configurations of anchor nodes. Meanwhile, a mobile node moves in this area along the true trajectory shown in Figure 3. The initial state of the mobile node is $\theta \left(0\right)={\left[\begin{array}{cccc} 0\;m & 20\;m & 1\;m/s & 0.5\;m/s \end{array}\right]}^{T}$, and the initial covariance matrix is $P(0)=I_{4}$. In order to simulate the complicated positioning environment, simulations are, respectively, taken under the condition that NLOS errors follow different distributions: Gaussian distribution, uniform distribution and exponential distribution. The propagation of NLOS errors between beacon nodes and mobile node is randomly generated with the probability $P_{nlos}$. The range measurements between the beacon nodes and the mobile node are sampled every 1 s, and 100 samples are sampled in total. The conventional EKF [5], REKF [7], IMM-EKF [19] and R-IMM [7] algorithms are compared with our algorithm. The parameter settings for simulation are presented in Table 1:

In this algorithm, we assume that the sensor noise variance $\sigma_{G}^{2}$ is known, then the measurement covariance matrix for the LOS model is chosen as $R^{*}{}_{1}\left(l\right)=\sigma_{G}^{2}I_{M}$ whereas that for NLOS model is chosen empirically to $R^{*}{}_{2}\left(l\right)=3\sigma_{G}^{2}I_{M}$, as in [7]. In addition, the other specific parameter settings are given as Table 2, Table 3 and Table 4 in Section 5.1.2.

The performance of the algorithm is evaluated through two indexes: cumulative distribution function (CDF) of the average localization error (ALE) and root mean square error (RMSE).

(63) $$RMSE=\sqrt{\frac{1}{L}\cdot \frac{1}{M_{c}}\sum_{a=1}^{M_{c}}\sum_{l=1}^{L}\left({\left({\hat{x}}_{a}\left(l\right)-x_{a}\left(l\right)\right)}^{2}+{\left({\hat{y}}_{a}\left(l\right)-y_{a}\left(l\right)\right)}^{2}\right)}$$

(64) $$ALE=\frac{1}{L}\sum_{l=1}^{L}\sqrt{{\left(\hat{x}(l)-x(l)\right)}^{2}+{\left(\hat{y}(l)-y(l)\right)}^{2}}$$

where *ALE* refers to the average distance deviation between the estimated position and the real position. (x(l),y(l)) and $\left(\hat{x}\left(l\right),\hat{y}\left(l\right)\right)$ represent the real position and estimated position respectively at time step *l*. L=100 denotes the number of samples, $M_{c}=1000$ is the Monte Carlo runs times.

#### 5.1.2. Simulation Results

In order to simulate a complex positioning environment, simulations are respectively taken under the condition that NLOS errors follow different distributions: Gaussian distribution, uniform distribution and exponential distribution. The robustness and positioning accuracy of the proposed algorithm with the change of the mean value of NLOS error, the standard deviation of NLOS error, the probability of NLOS error, and the number of beacon nodes are explored based on the comparative scheme designed. Simulation results are shown and evaluated in Section 5.1.2.1, Section 5.1.2.2 and Section 5.1.2.3 in this part.

##### 5.1.2.1. Gaussian Distribution

We first assume NLOS error and sensor noise follow Gaussian distribution $N\left(\mu_{nlos},\sigma_{nlos}^{2}\right)$ and $N\left(0,\sigma_{G}^{2}\right)$ respectively. Since NLOS error is always greater than zero, $\left|N\left(\mu_{nlos},\sigma_{nlos}^{2}\right)\right|$ is further used to simulate NLOS error. The default parameters in this simulation are displayed in Table 2.

**Figure 4:**
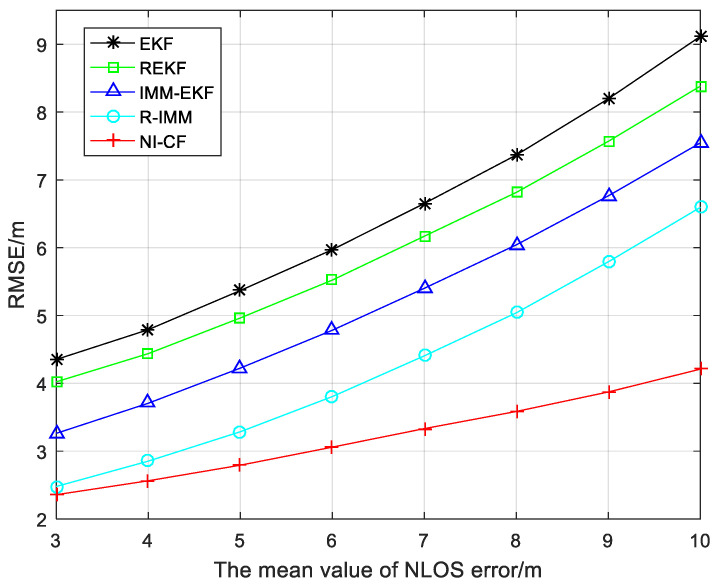
The root mean square error (RMSE) versus the mean value of NLOS error.

To better illustrate the robustness and positioning accuracy of the proposed algorithm as the mean value of NLOS error $\mu_{nlos}$ changes, Figure 4 shows the RMSE results with $\mu_{nlos}$ changing from 3 to 10. As the increase of $\mu_{nlos}$, the RMSE of five algorithms for comparison all have a trend of increasing. But we can observe that the positioning accuracy of the proposed algorithm is always the highest and decreases more slowly as $\mu_{nlos}$ increases, which shows stronger robustness. The comparison table of average RMSE value is shown as Table 3.

**Figure 5:**
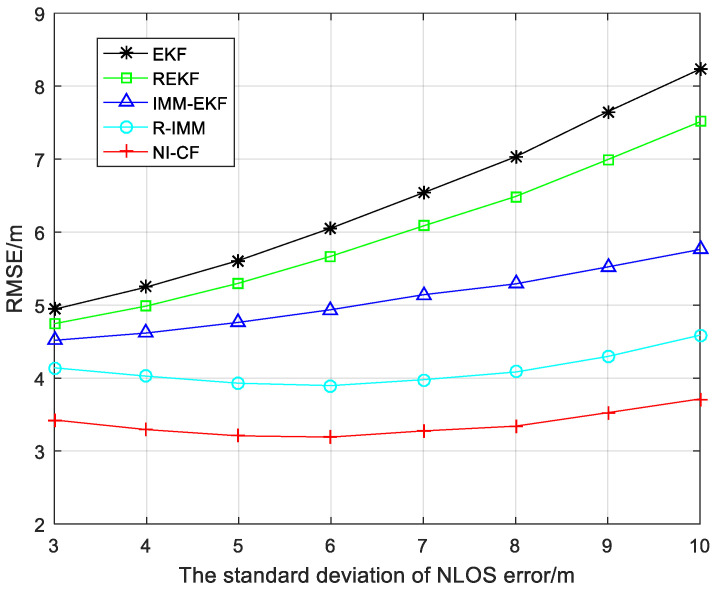
The RMSE versus the standard deviation of NLOS error.

Figure 5 shows the RMSE of each algorithm as the standard deviation of NLOS error $\sigma_{nlos}$ changes from 3 to 10. When the standard deviation of NLOS error increases, the performance of five algorithms all declines. Compared with other algorithms, the proposed algorithm always maintains the optimal positioning accuracy and the RMSE only increases slightly with the increase of $\sigma_{nlos}$.

**Figure 6:**
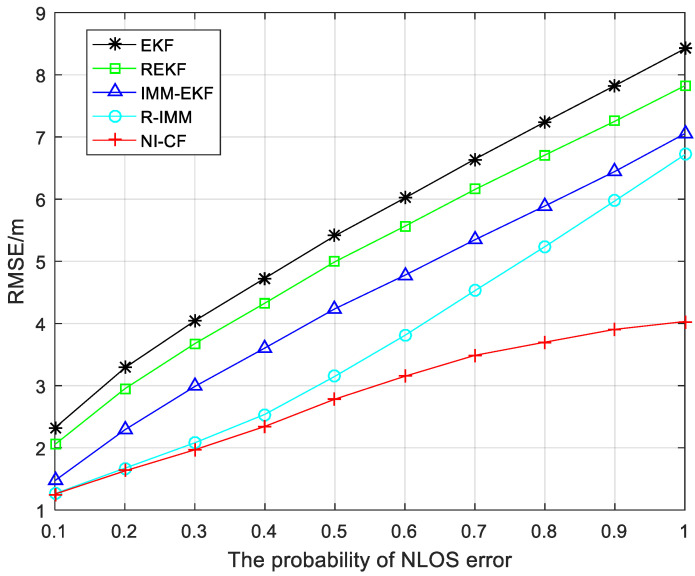
The RMSE versus the probability of NLOS error.

The positioning accuracy and robustness of the proposed algorithm when the probability of NLOS error varies from 0.1 to 1 are shown in Figure 6. The greater the NLOS error probability, the greater the RMSE of five algorithms, and we can notice that in the process of NLOS probability increasing from 0.1 to 1, the positioning accuracy of the proposed algorithm is higher than the other four algorithms, and the greater the probability of NLOS error, the more obvious the advantages of the proposed algorithm. The proposed algorithm increases the average positioning accuracy to 2.8259 m, while the average positioning accuracy of the EKF, REKF, IMM-EKF and R-IMM is 5.5937 m, 5.1506 m, 4.4104 m, and 3.6967 m, respectively.

**Figure 7:**
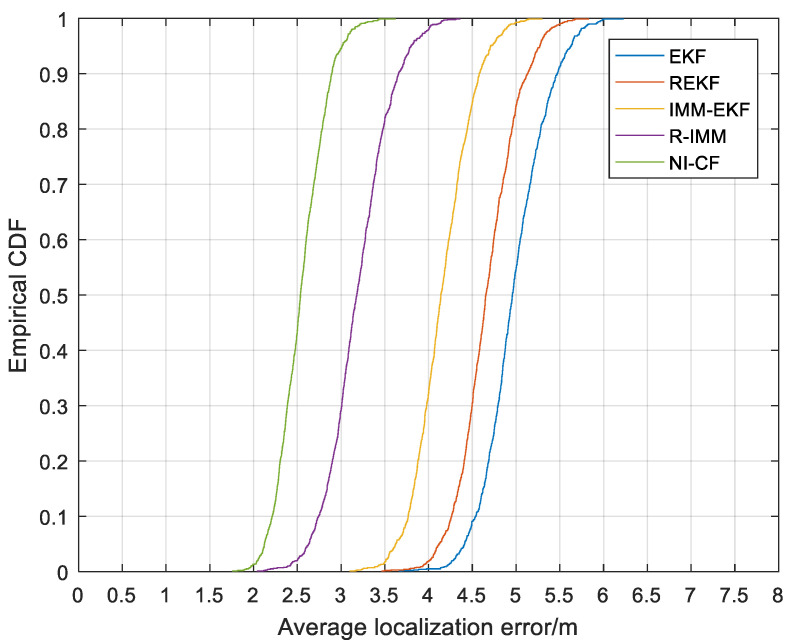
The cumulative distribution function (CDF) versus the average localization error (ALE).

Figure 7 is the CDF of ALE. From the figure, we can see that the 90%-quantile of ALE of the proposed algorithm is about 2.9 m, while the corresponding values of EKF, REKF, IMM-EKF and R-IMM are about 5.5 m, 5.2 m, 4.6 m and 3.7 m respectively.

##### 5.1.2.2. Uniform Distribution

We assume that that NLOS error and sensor noise follow uniform distribution U(min,max) and Gaussian distribution $N\left(0,\sigma_{G}^{2}\right)$ respectively. The default parameters in this simulation are displayed in Table 4.

**Figure 8:**
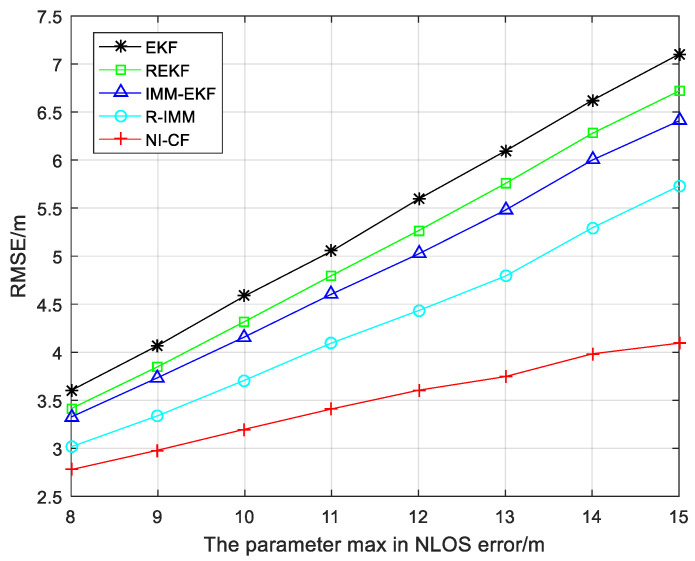
The RMSE versus the parameter max in NLOS error.

The RMSE result of each algorithm with the parameter max changing from 8 to 15 is described in Figure 8. Different from the trend of significantly increasing RMSE of the other four algorithms, the RMSE of the proposed algorithm increases slightly when the parameter max increases from 8 to 15, which means stronger robustness. The average RMSE values of different algorithms are shown in Table 5.

**Figure 9:**
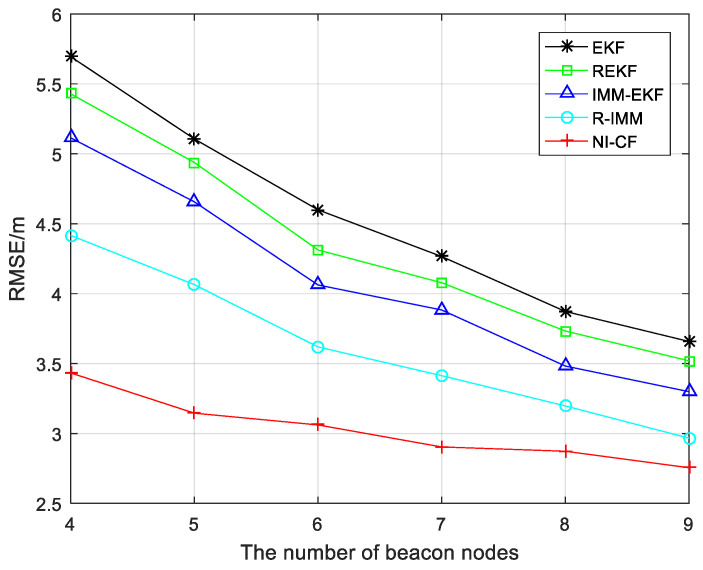
The RMSE versus the number of beacon nodes.

Figure 9 explores the influence of different beacon node numbers on positioning accuracy. The RMSE of each algorithm increases when the number of beacon nodes gradually decrease from 9 to 4, which shows that more beacon nodes are helpful to improve the positioning accuracy. The positioning accuracy of the proposed algorithm does not decrease significantly with the decrease of the number of beacon nodes, which means that the proposed algorithm has less dependence on the number of beacon nodes participating in positioning.

##### 5.1.2.3. Exponential Distribution

We assume that NLOS error and sensor noise follow exponential distribution E(λ) and Gaussian distribution $N\left(0,\sigma_{G}^{2}\right)$ respectively. The default parameters in this simulation are displayed in Table 6.

**Figure 10:**
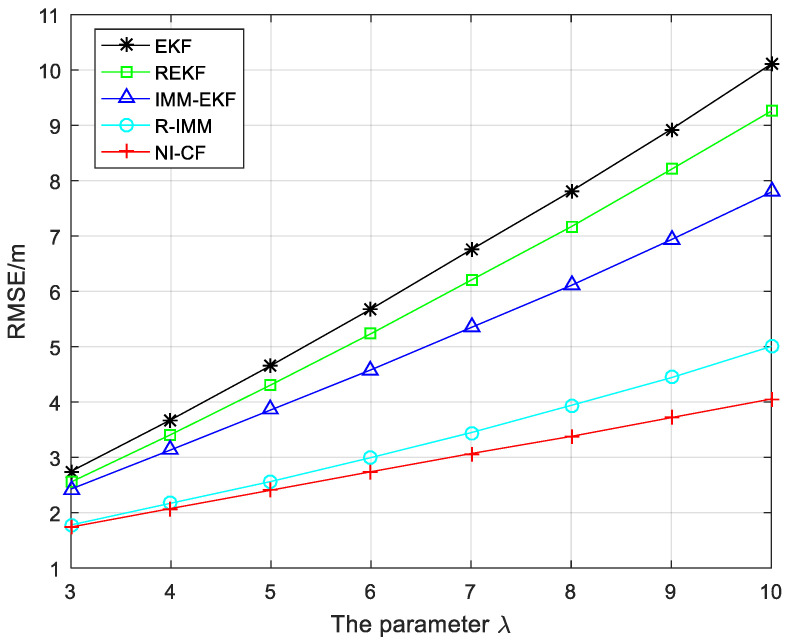
The RMSE versus the parameter λ.

The RMSE of each algorithm with the parameter λ in exponential distribution changing from 3 to 10 is shown in Figure 10. With the increase of parameter λ, the RMSE of each algorithm increases. By contrast with the other three algorithms, the RMSE of R-IMM and the proposed algorithm increases slightly. Meanwhile, it can be observed that the positioning accuracy of the proposed algorithm is significantly higher than that of the other four algorithms.

**Figure 11:**
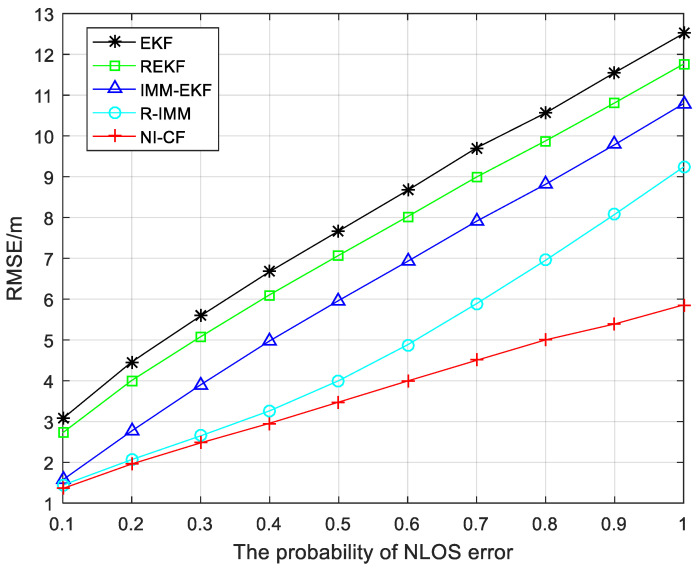
The RMSE versus the probability of NLOS error.

To describe the robustness of the proposed algorithm as the NLOS error probability $P_{nlos}$ changes, Figure 11 shows the RMSE results when $P_{nlos}$ varying from 0.1 to 1. The larger $P_{nlos}$, the larger the RMSE of the five algorithms for comparison. This is reasonable since the NLOS situation is more serious, and the proposed algorithm performs better than other algorithms.

**Figure 12:**
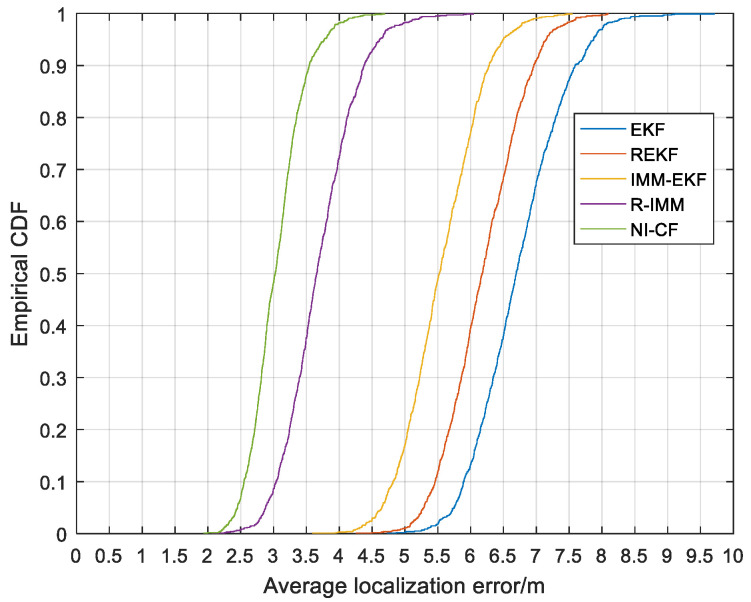
The CDF versus the ALE.

From Figure 12, we can see that the 90%-quantile of ALE of the proposed algorithm is about 3.5 m, while that of ALE of EKF, REKF, IMM-EKF and R-IMM is about 7.6 m, 7.0 m, 6.3 m and 4.4 m respectively.

### 5.2. Experiment

#### 5.2.1. Experimental Environment

**Figure 13:**
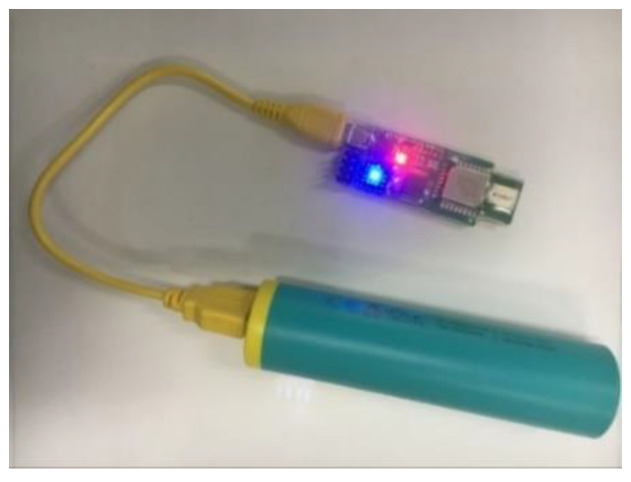
The ultra-wideband (UWB) node connected with power supply.

In order to test the performance of the proposed algorithm, we conducted a localization experiment in an indoor environment. The signal transmission between mobile node and beacon nodes was based on ultra-wideband (UWB) technology. UWB technology has been widely used in indoor positioning in recent years because of its low complexity and high positioning accuracy. The principle of UWB ranging is to approximate the distance between nodes based on the two-way propagation time of signals. In this experiment, both the mobile node and the beacon nodes adopted UWB node. The difference was that the mobile node was connected with the power supply as shown in Figure 13, and was held by a pedestrian during the movement, while a beacon node was connected with a computer to transmit the collected data to the computer.

**Figure 14:**
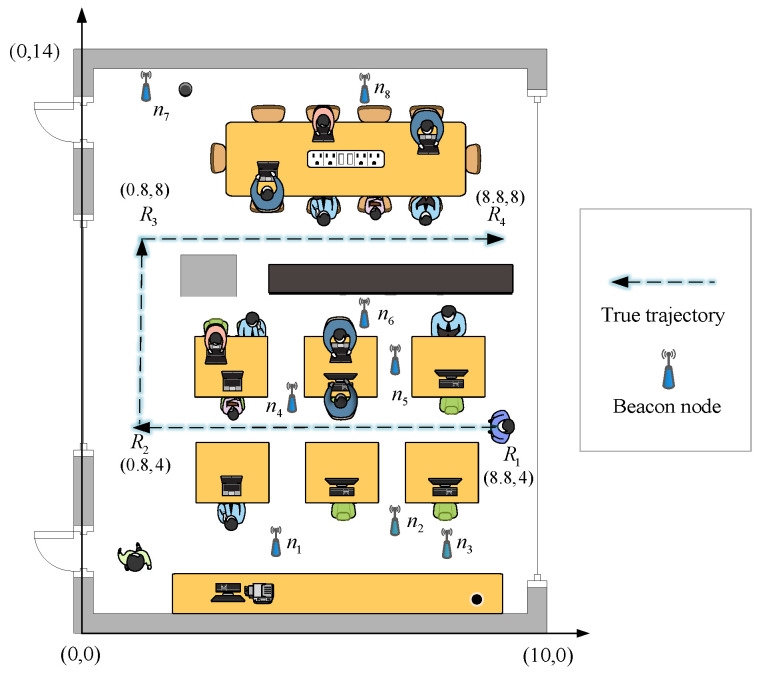
The experimental scene.

The experimental scene is depicted in Figure 14. In the indoor localization area of 10 m * 14 m, the coordinates of eight beacon nodes are $n_{1}(4,0.8)$, $n_{2}(7.2,1.6)$, $n_{3}(8,0.8)$, $n_{4}(4.8,4.8)$, $n_{5}(7.2,5.6)$, $n_{6}(6.4,6.4)$, $n_{7}(0.8,12.8)$, $n_{8}(6.4,12.8)$. The mobile node moves at a speed of 0.8 m/s along the true trajectory in the figure from the initial position $R_{1}(8.8,4)$ until it reaches $R_{4}(8.8,8)$. To avoid ground reflection, all nodes are placed 1.2 m above the ground. The sampling frequency is 1 Hz. Every 80 cm, one sampling point is recorded and there are 26 sampling points in the whole true trajectory. Twenty measurements are collected at every sampling point, and the average of 20 measurements at every sampling point are utilized as the measurement distance. The parameter settings for the experiment were the same as those in the simulation.

#### 5.2.2. Experimental Results

**Figure 15:**
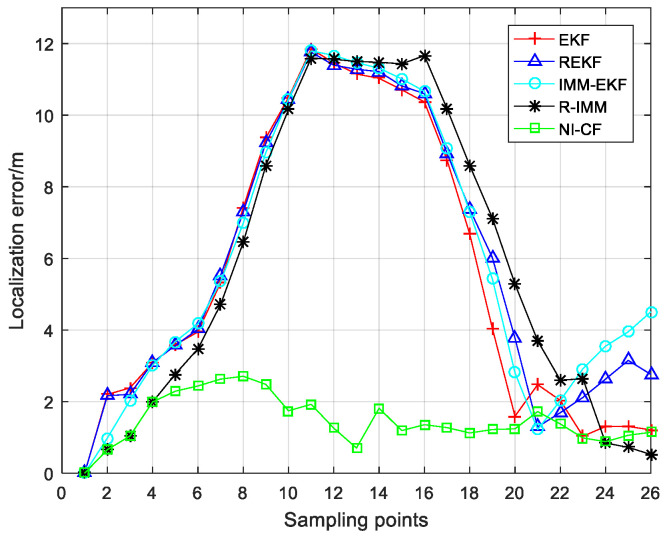
The localization error at each sampling point.

**Figure 16:**
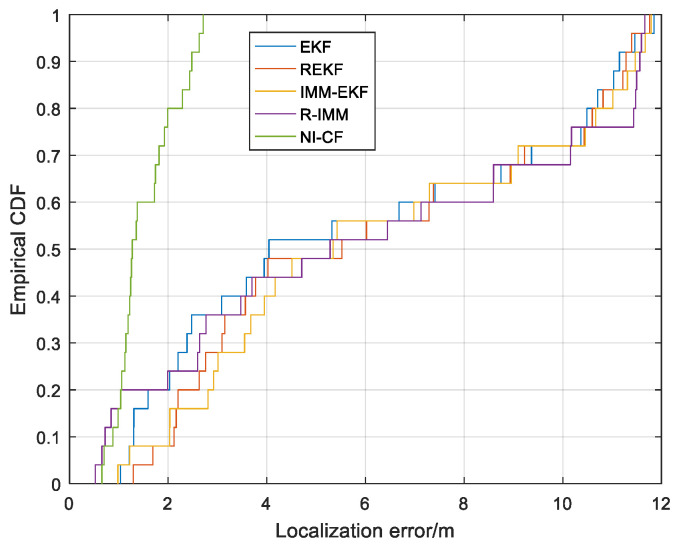
The CDF versus the localization error.

The localization error at each sampling point is illustrated in Figure 15. We can find that the localization error of the proposed algorithm is almost always the smallest. Figure 16 illustrates the CDF of localization error. From the figure, we can see that the 90%-quantile of the localization error of the proposed algorithm is only about 2.5 m, while that of the other four algorithm exceeds 11 m, which shows that the proposed algorithm can effectively suppress the negative effects of NLOS errors on positioning accuracy.

#### 5.2.3. Computation Time Comparison

Table 7 shows the running time of each algorithm. The five algorithms are coded using MATLAB R2016a and tested on a Windows 10 Professional workstation with Intel(R) Core (TM) i7-8700 @ 3.20GHz and 8.00GB RAM. Although NI-CF consumes more time, the time for a single processing is still far less than the interval between single sample (the sampling frequency is 1 Hz). Therefore, the algorithm can be applied for online tracking.

## 6. Conclusions

This paper proposes a robust localization algorithm based on NLOS identification and classification filtering for a hybrid LOS and NLOS environment. Firstly, an NLOS identification strategy is proposed to detect the severity of NLOS, and then the NLOS situation is further divided into mild NLOS and severe NLOS, which is beneficial to the subsequent classification filtering. After that, we performed classification filtering to obtain respective position estimates for different NLOS situations. For the LOS case, the EKF algorithm was applied to generate accurate positioning results. For the NLOS case, REKF algorithm and the proposed severe NLOS mitigation algorithm based on LOS reconstruction were employed to filter out mild NLOS noise and severe NLOS noise, respectively. Through NLOS identification and classification filtering, the proposed algorithm could deal better with various complex positioning environments and had stronger robustness. Finally, the IMM algorithm was employed to obtain the final positioning result by weighing the position estimation of LOS and NLOS, which further improved the positioning accuracy and robustness. Simulation and experiments showed that the proposed algorithm outperformed EKF, REKF, IMM-EKF, R-IMM in the hybrid LOS and NLOS environment. In addition, under the unfavorable conditions of fewer beacon nodes participating in the positioning and higher NLOS probability, the performance of the algorithm did not drop significantly, which shows stronger robustness. In the future, we need to further improve the positioning accuracy when the number of nodes participating in the positioning has enough redundancy.

## Figures and Tables

**Table 1:** The parameter settings for simulation.

Meaning	Values
the warning probability of the validation gate	$P_{FA}=0.01$
the initial mode probability	$\mu_{1}(0)=0.5$ $\mu_{2}(0)=0.5$
the driving noise covariance matrix	$Q\left(l\right)=I_{2}$
the measurement covariance matrix	$R^{*}{}_{1}\left(l\right)=\sigma_{G}^{2}I_{M}$ $R^{*}{}_{2}\left(l\right)=3\sigma_{G}^{2}I_{M}$
the clipping points of location score function	$c_{1}=1.5$ $c_{2}=3$

**Table 2:** The default parameters for Gaussian distribution.

Symbol	Meaning	Default Values
$P_{nlos}$	NLOS probability	0.5
$\left|N\left(\mu_{nlos},\sigma_{nlos}^{2}\right)\right|$	NLOS error	$\left|N\left(6,6^{2}\right)\right|$
*M*	Number of beacon nodes	6
$N\left(0,\sigma_{G}^{2}\right)$	Sensor noise	$N\left(0,1^{2}\right)$

**Table 3:** Comparison table of average RMSE value.

Algorithm	The Average RMSE Value/m
NI-CF	3.2217
R-IMM	4.2818
IMM-EKF	5.2163
REKF	5.9851
EKF	6.4764

**Table 4:** The default parameters for uniform distribution.

Symbol	Meaning	Default Values
$P_{nlos}$	NLOS probability	0.5
U(min,max)	NLOS error	U(0,12)
*M*	Number of beacon nodes	6
$N\left(0,\sigma_{G}^{2}\right)$	Sensor noise	$N\left(0,1^{2}\right)$

**Table 5:** The average RMSE values of different algorithms.

Algorithm	The Average RMSE Value/m
NI-CF	3.4745
R-IMM	4.3012
IMM-EKF	4.8421
REKF	5.0497
EKF	5.3401

**Table 6:** The default parameters for exponential distribution.

Symbol	Meaning	Default Values
$P_{nlos}$	NLOS probability	0.5
E(λ)	NLOS error	E(8)
M	Number of beacon nodes	6
$N\left(0,\sigma_{G}^{2}\right)$	Sensor noise	$N\left(0,1^{2}\right)$

**Table 7:** Running time of each algorithm.

Algorithm	Running Time/s
EKF	0.0020
REKF	0.0025
IMM-EKF	0.0028
R-IMM	0.0046
NI-CF	0.0111
